# Irregular seasonality of respiratory syncytial virus infection persists in 2023 in Osaka, Japan

**DOI:** 10.1016/j.ijregi.2024.100442

**Published:** 2024-09-05

**Authors:** Takeshi Miyama, Kensaku Kakimoto, Yasutaka Yamanaka, Yoko Nishida, Nobuhiro Iritani, Kazushi Motomura

**Affiliations:** 1 Epidemiology Section, Division of Public Health, Osaka Institute of Public Health, Osaka, Japan; 2 Emergency Preparedness and Response Section, Division of Public Health, Osaka Institute of Public Health, Osaka, Japan

**Keywords:** Epidemiology, Japan, Mathematical model, Periodicity, Respiratory syncytial virus infections, Seasonal variation

## Abstract

**Objectives:**

Owing to the nonpharmaceutical interventions against COVID-19, respiratory syncytial virus (RSV) infection was nearly absent in 2020. An unusual epidemic size and irregular seasonal pattern were observed worldwide in 2021. In Osaka, Japan, after disrupting the regular pattern of RSV infection dynamics (before the COVID-19 pandemic, RSV epidemics typically start in summer and peak around fall), the epidemic size of RSV infection returned to normal in 2022. However, the epidemic onset timing remained irregular in 2022 and 2023. This study investigated whether the onset of the RSV infection epidemic in 2023 was predictable using previous seasonal patterns.

**Methods:**

The weekly number of RSV infection cases obtained from sentinel pediatric sites between 2007 and the 15th week of 2023 was modeled using the time series susceptible-infected-recovered model. Forecasting of the remainder of 2023 was conducted based on estimated transmission parameters.

**Results:**

None of the estimated transmission rates from previous years successfully forecast the epidemic onset in 2023. Only the transmission rate estimated in the early part of 2023 captured the trend for that year, indicating irregular seasonal transmission rates.

**Conclusions:**

It is still hard to forecast RSV epidemics because of the changed landscape due to the COVID-19 pandemic. The seasonality of RSV infection dynamics has not returned to pre-pandemic level in 2023. Cautious attention to future RSV dynamics in Japan is warranted because further changes may occur in the near future.

## Highlights

• During the COVID-19 pandemic, irregular respiratory syncytial virus patterns were observed worldwide.
• Winter respiratory syncytial virus seasons occurred in 2023 in various countries, as seen before the pandemic.
• In Osaka, Japan, the onset timing of epidemics remained irregular in 2023.

## Introduction

There have been several nonpharmaceutical interventions against COVID-19, such as physical distancing and mask wearing. Thus, the absence of an epidemic on several respiratory infectious diseases, such as influenza and respiratory syncytial virus (RSV) infection, was observed worldwide during the pandemic in 2020, including Japan. This was followed by unusual epidemic sizes and irregular annual seasonal patterns [[1], [2], [3]]. Knowledge about the size and onset timing of an epidemic is important for preventive measures for reducing disease burden, which include influenza vaccination or monoclonal antibody palivizumab treatment for RSV infection. New options for RSV control, such as nirsevimab (another type of monoclonal antibody), maternal vaccines to protect infants, and vaccination for the elderly, are now becoming available worldwide, including in Japan. For effective prevention, understanding the seasonality of RSV has become increasingly important recently. In Osaka, Japan, after disrupting the regular pattern of RSV infection dynamics caused by the COVID-19 pandemic, the annual incidence of RSV infection returned to normal in 2022 (the reported numbers of cases were as follows: 12,463, 1123, 16,013, and 11,861 in 2019, 2020, 2021, and 2022, respectively). However, the timing of epidemic onset was still irregular in 2022 and 2023 in Japan (Figure S1). In addition, in several countries in the northern hemisphere, the winter RSV seasons are in 2022/2023 and 2023/2024, as observed in the pre-pandemic period [4,5]. The current study investigated whether the onset of the RSV infection epidemic in Osaka, Japan in 2023 was predictable using a mathematical model that incorporates the historical pattern of seasonal transmissibility (i.e. time-varying transmission rate).

## Materials and methods

The weekly number of reported RSV infection cases aged under 15 years and the number of births in Osaka Prefecture were used to model the RSV dynamics between 2007 and 2023. RSV infection cases are reported under a pediatric sentinel surveillance system in the National Epidemiological Surveillance of Infectious Diseases Program in Japan. Approximately 3000 hospitals and clinics are registered as pediatric sentinel sites in Japan (in Osaka, 190 hospitals and clinics). The configuration of a pediatric sentinel site is set at a ratio of 30,000-50,000. We retrieved data on the weekly number of reported cases of RSV infection from the National Epidemiological Surveillance of Infectious Diseases system [6]. The report is based on laboratory diagnosis using enzyme immunoassays and/or nucleic acid amplification tests for nasal/throat swab specimens; the severity of cases, such as hospitalization status, is not included in the surveillance data. The number of births until 2022 was obtained from the vital statistics of Japan [7]. We assumed that the number of births in 2023 was similar to that in 2022. The data used in this study did not include any personal information and are publicly available. Therefore, ethical approval and consent are not required.

We modeled the time series of the weekly number of RSV infection cases from the 1st week of 2007 to the 15th week of 2023 using the time series susceptible-infected-recovered (TSIR) model [8]. In the TSIR models, the reporting rate is considered as the slope from the regression of the cumulative number of births on the cumulative number of reported cases under the assumption that all newborns are infected. We reconstructed the number of infected individuals, $I_{t}$, and susceptible individuals, $S_{t}$, by estimating the reporting rate and time-varying transmission rate, $\beta_{t}$, assuming it is constant for 8 weeks. The supplementary information shows the details of the model description. Using the previously mentioned model, we forecast the weekly incidence in 2023 by assuming that the transmission rate in 2023 was similar to that in 2019, 2021, and 2022. We also created a scenario assuming the transmission rate in 2023 as the constant $\beta_{t}$ as of week 15 in 2023 (i.e. the latest $\beta_{t}$ estimated, Supplementary Figure S2). The cut-off time, week 15 of 2023, was chosen somewhat arbitrarily when the epidemic onset became evident. We checked whether the cut-off time affects the forecasting results.

## Results

**Figure 1:**
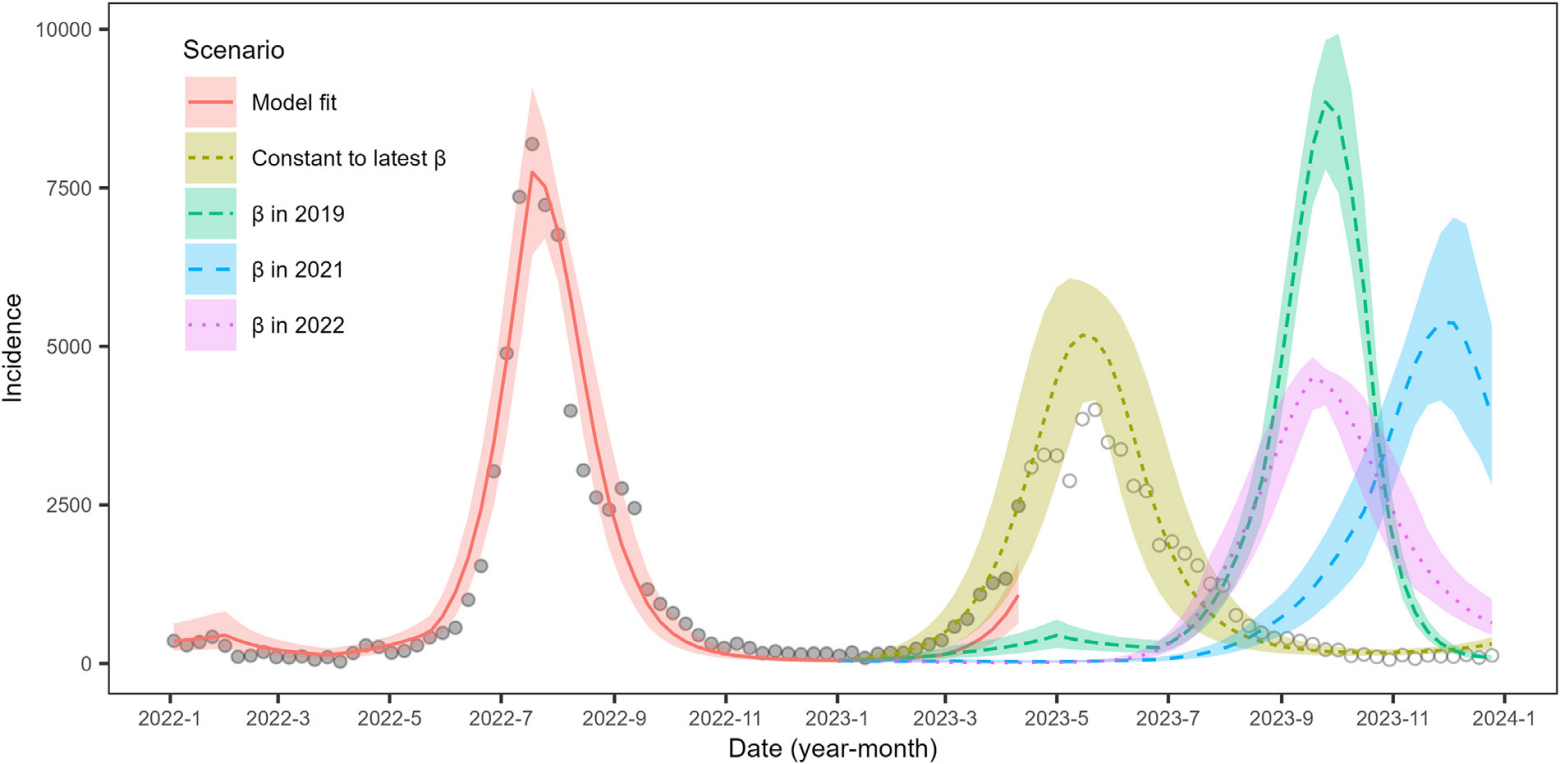
Respiratory syncytial virus infection incidence estimated using the time series susceptible-infected-recovered model in 2022 and 2023 in Osaka, Japan. The model fit was done using the reported number of cases by week 15 in 2023. Forecasting in 2023 was conducted using the following scenario: (a) the transmission rate $\beta_{t}$ in 2023 was assumed to be the constant to the latest value estimated (constant to latest β). See supplementary information for the value of $\beta_{t}$. (b) The same level of $\beta_{t}$ in 2019 (β in 2019), (c) β in 2021, and (d) β in 2022 were assumed. The lines and shades are the median and 95% confidence interval estimations. The filled dots are the numbers of incidences used for the model fit, and the open dots are those not used. They are calculated as the number of reported cases, $C_{t}$, divided by the reporting rate estimated, $\delta_{t}$ ($C_{t}/\delta_{t}$).

The number of susceptible individuals and incidences in Osaka from 2007 to week 15 in 2023 was reconstructed using the TSIR model (Figure S2). Notably, the number of susceptible individuals reached the highest level during the study period in 2020 because of the intervention against COVID-19. The estimated $\beta_{t}$ was unusually lower than the rest of the period in the early 2020, followed by the wide confidence interval caused by uncertainty affected by the small number of reported cases. Increased susceptibility has led to a larger and longer epidemic with an irregular seasonality than regular seasons (pre-pandemic period). That is, the common RSV infection season in Osaka is from summer to fall and winter [9]. However, in 2021, the epidemic period started from the beginning of the year. The estimated transmission rate in 2023 was high, and epidemic onset was observed from the beginning of the year. The scenario of the latest high $\beta_{t}$ estimation could only capture the observed transmission dynamics in RSV infection in 2023 (constant to the latest β in Figure 1 and Supplementary Figures S3 and S6). The rest of the scenario using $\beta_{t}$ in previous years failed to capture the epidemic onset in 2023.

## Discussion

The cyclicity of infectious disease dynamics is influenced by the dynamics of susceptible individuals (the recruitment and removal of susceptible individuals, e.g. births, waning from immunity, infection, and/or deaths). Moreover, they are essential for understanding the long-term dynamics [10]. They may remain important in analyzing the short-term future dynamics of infectious diseases, as presented in this study. Therefore, a precise reconstruction of the number of susceptible individuals can improve the capability of future epidemic forecasting. Given the reconstructed number of susceptible individuals estimated in the current study, the transmission rate in 2023 was relatively higher than that in other years, which could be one of the reasons for the early epidemic onset of RSV infection observed in 2023. The rationale for the high transmission rate in 2023 is unclear. However, a high RSV prevalence in the 2022/2023 season observed worldwide in winter [4,5] and the relaxed COVID-19 interventions that induce a higher inflow of virus carriers from abroad and that activate internal human interaction may have contributed to the high transmission rate. The possibility of virus-virus interactions and viral interference was not considered in this study. Further analysis is required to investigate the factors influencing the high transmission rate. Notably, the following issues might influence the precision of the reconstruction. First, the reporting rate during the COVID-19 pandemic can be increased with a higher frequency of tests for differential diagnosis. The respiratory pathogen multi-screening for suspicious COIVD-19 cases, which includes RSV screening, was covered by health insurance during the pandemic. Caution is needed in case the actual increase in the reporting rate was not captured by the analysis. The number of infections could be overestimated, which could lead to an underestimation of the number of susceptible individuals and an overestimation of the transmission rate. In this study, the smoothing spline regression was used to estimate the reporting rate, which captures its time-dependency. In addition, in the sensitivity analysis, we increased the degree of freedom (from 5 to 8) of the smoothing spline to reflect the recent trend of reporting rate during the pandemic and obtained consistent results (Supplementary Figures S4 and S5). Second, we assumed that the birth rate in 2023 was the same as that in 2022. However, it has a declining trend, particularly, during the pandemic. Therefore, the recruitment rate in susceptible individuals could have been overestimated, which causes an underestimation of the reporting rate. It should be noted that the data used in this study were obtained from sentinel pediatric sites and may not be representative of the entire population. However, considering that the age-dependent timing of epidemics is expected to be similar within the same area, including only data from pediatric sites may have had a minimal impact on the generalizability of the timing to the entire population in Osaka.

After disrupting the regular pattern of RSV infection dynamics due to COVID-19 interventions, we investigated whether the onset of the RSV infection epidemic in Osaka, Japan, in 2023 was predictable and revealed that it is still hard to forecast RSV epidemics. The seasonality of RSV infection dynamics has not returned to the pre-pandemic level in 2023. Because capturing all aspects that influence epidemic cyclicity (e.g. the balance between the number of susceptible individuals and infections) is challenging, monitoring the dynamics of recent infections is important to understand the onset of future epidemics. Owing to the relaxed interventions, human interaction from abroad and within Japan has been returning to the pre-pandemic level. We should pay cautious attention to the future RSV dynamics in Japan because it could be influenced by the prevalence worldwide and further change might be expected in the near future.

## Declarations of competing interest

The authors have no competing interests to declare.

## Appendix Supplementary materials

Image, application 1
