# Supplementary material for: Irregular seasonality of respiratory syncytial virus infection persists in 2023 in Osaka, Japan
Source: IJID Reg. 2024 Sep 5;13:100442. doi: 10.1016/j.ijregi.2024.100442 (PMC11462021; doi:10.1016/j.ijregi.2024.100442)
Supplement: Supplementary file 1 [file mmc1.docx]

Supplementary information for the manuscript entitled “**Irregular seasonality of respiratory syncytial virus infection persists in 2023 in Osaka, Japan”**

**Takeshi Miyama^1)^, Kensaku Kakimoto^2)^, Yasutaka Yamanaka^2)^, Yoko Nishida^2)^, Nobuhiro Iritani^2)^, Kazushi Motomura^1)^**

^1)^ Epidemiology Section, Division of Public Health, Osaka Institute of Public Health, Japan

^2)^ Emergency Preparedness and Response Section, Division of Public Health, Osaka Institute of Public Health, Japan

# Supplementary methods

## Mathematical model

We modeled the time series of the weekly number of RSV infection cases from the 1st week of 2007 to the 15th week of 2023 using the time-series susceptible-infected-recovered (TSIR) model ^[1–3]^. The number of susceptible individuals under a discrete time SIR model with a time step of generation time (set as 1 week for RSV infection) can be written as follows:

$\begin{aligned} \begin{aligned} S_{t+1}=S_{t}+B_{t}-I_{t}\#\# \end{aligned},\#\left( 1 \right) \end{aligned}$where $S_{t}$ is the number of susceptibles, $I_{t}$ and $B_{t}$ are the number of infected individuals and births at time *t*, respectively*.* The expected number of infected cases at each time step can also be written as

$$\begin{aligned} \begin{aligned} I_{t+1}=\beta_{t}\frac{S_{t}}{N}I_{t}^{\alpha}\#\#\# \end{aligned},\#\left( 2 \right) \end{aligned}$$

where $\beta_{t}$ is a time-varying transmission rate which is 8 weeks constant, and $\alpha$ is a constant that captures nonhomogeneous mixing and the discretization of a continuous time process, and $\alpha$ is assumed to be 0.97, referring to ^[4]^. We assumed that the number of RSV infections follows a Poisson distribution (i.e., $\frac{C_{t}}{\delta_{t}}\sim Poisson\left( I_{t} \right)$), where $C_{t}$ is the number of cases reported through the reporting system and $\delta_{t}$ is the reporting rate that accounts for all infections that were not captured by the system. By taking the logarithm, Equation (2) becomes

$$\begin{aligned} \ln(I_{t+1})=\ln(\beta_{t})+\alpha\ln(I_{t})+\ln(S_{t})-\ln\left( N \right),\#\left( 3 \right) \end{aligned}$$

which is the Poisson regression form of $\ln(I_{t+1})$ to estimate $\beta_{t}$ with $\alpha\ln(I_{t})$, $\ln(S_{t})$, and $\ln\left( N \right)$ as offsets. To conduct this regression analysis, the time-varying number of susceptible, $S_{t}$ is required. Using Equation (1), its reconstruction can be rewritten as follows:

$$\begin{aligned} S_{t}=\bar{S}+D_{0}+\sum_{k=0}^{t} B_{k}-\sum_{k=0}^{t} \frac{C_{k}}{\delta_{k}},\#\left( 4 \right) \end{aligned}$$

where $\bar{S}$ is the mean number of susceptibles, $D_{0}$ is the deviation of susceptibles at time 0 from $\bar{S}$ (i.e., the number of susceptible at time 0, $S_{0}=\bar{S}+D_{0}$), and $B_{k}$ is the number of newborns at time *t*. Equation (4) can be written in the form of regression of the cumulative number of births on the cumulative number of infections as follows:

$$\begin{aligned} \sum_{k=0}^{t} B_{k}=-D_{0}+\sum_{k=0}^{t} \frac{C_{k}}{\delta_{t}}+D_{t},\#\left( 5 \right) \end{aligned}$$

where $D_{t}$ is the deviation of susceptible at time *t* from $\bar{S}$ (i.e., $S_{t}=\bar{S}+D_{t}$), which is the residual from the regression. We can obtain the estimation of $\delta_{t}$ and $D_{t}$. We used the smoothing spline method with 5 degrees of freedom for the regression considering that reporting rates may vary over time ^[5]^ and regarded the first derivative (slope) of the smoothing spline as the reporting rate. We also conducted a sensitivity analysis for $\delta_{t}$ estimation with an increased degree of freedom (from 5 to 8) to sensitively reflect the recent trend in the reporting rate during the pandemic. $\bar{S}$ can be estimated from the regression of Equation (3) by setting it as a variable and profiling its likelihoods. For $\bar{S}$ estimation, we applied a 2-week constant seasonal (periodic) transmission rate for $\beta_{\tau}$ {$\tau=1,2,\ldots,26$} instead of a time-varying transmission rate, $\beta_{t}$ to constrain the number of parameters to be estimated. We used the time series data between 2007 and 2019 to estimate $\bar{S}$ because the unusual seasonality was observed in RSV dynamics during the COVID-19 pandemic. Finally, using the estimated parameters, $I_{t}$ and $S_{t}$ were reconstructed. The parametric bootstrap method with 1,000 iterations was used to calculate the 95% confidence interval ^[6]^.

# Supplementary figures


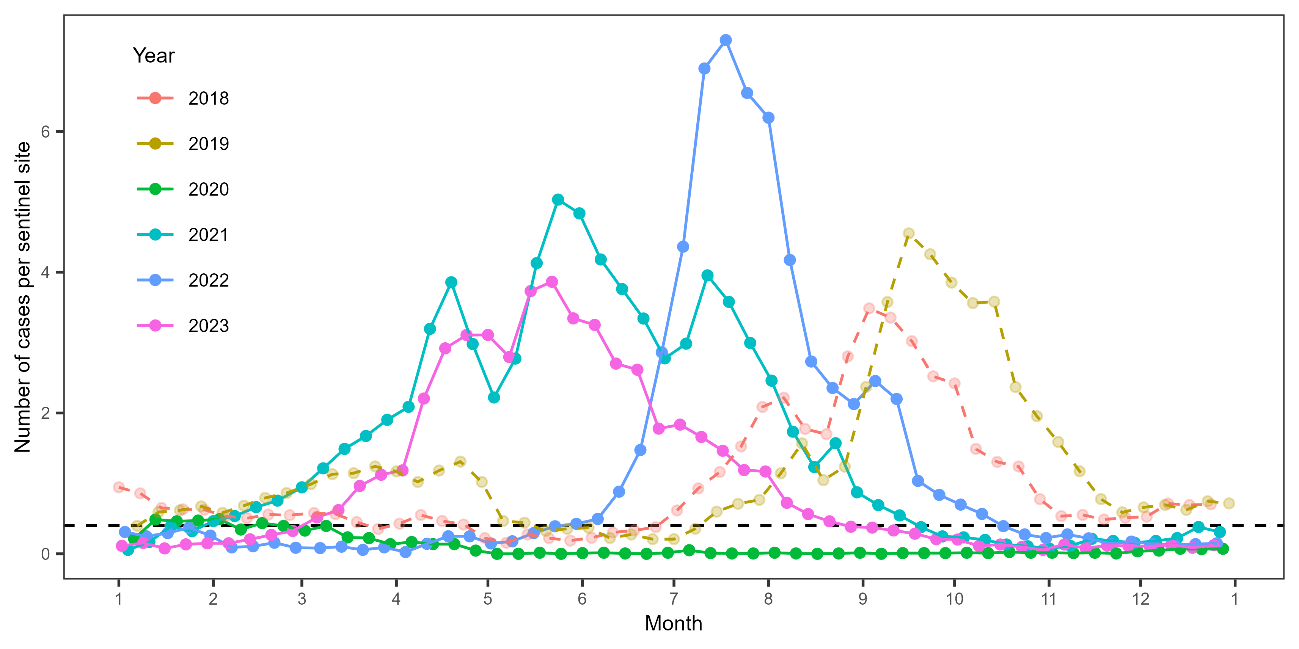


**Figure S1 Number of reported cases per pediatric sentinel site for RSV dynamics in Osaka, Japan, from 2018 to 2023**. Epidemic curves before the COVID-19 pandemic are shown with dashed lines, while those during and after the pandemic are shown with solid lines. The horizontal dashed line at 0.4 cases per site indicates the onset level of the epidemic estimated by ^[7]^.


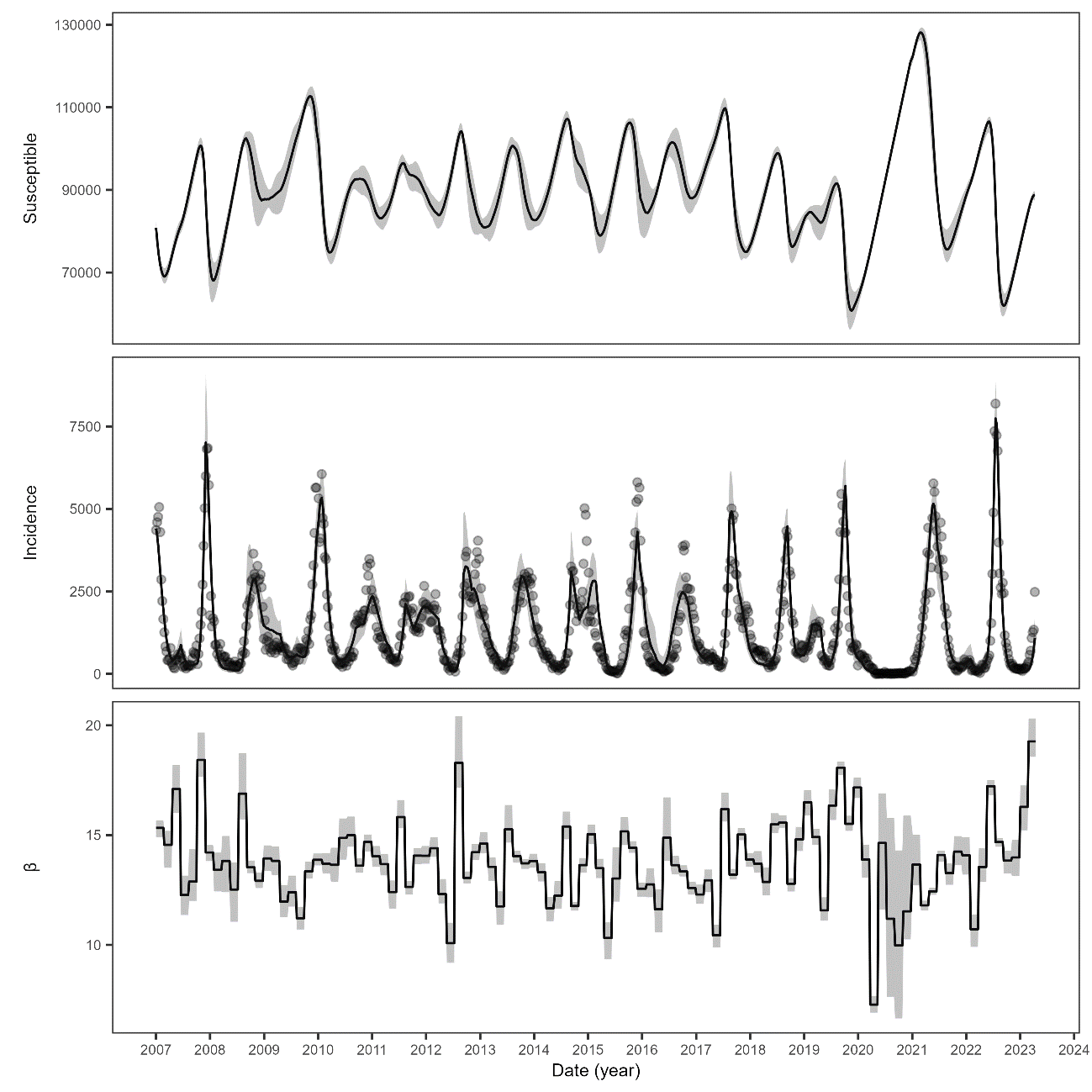


**Figure S2 Reconstructed number of susceptible individuals and incidences (**$\boldsymbol{S}_{\boldsymbol{t}}$ **and** $\boldsymbol{I}_{\boldsymbol{t}}$**) and estimated transmission rate,** $\boldsymbol{\beta}_{\boldsymbol{t}}$ **using the time-series susceptible-infected-recovered (TSIR) model from 2007 to week 15 in 2023 in Osaka, Japan.** The lines and shades represent the median and 95% confidence interval estimations. The filled dots are the numbers of incidences used for the model fit. They are calculated as the number of reported cases, $C_{t}$ over the reporting rate estimated, $\delta_{t}$ ($C_{t}/\delta_{t}$).


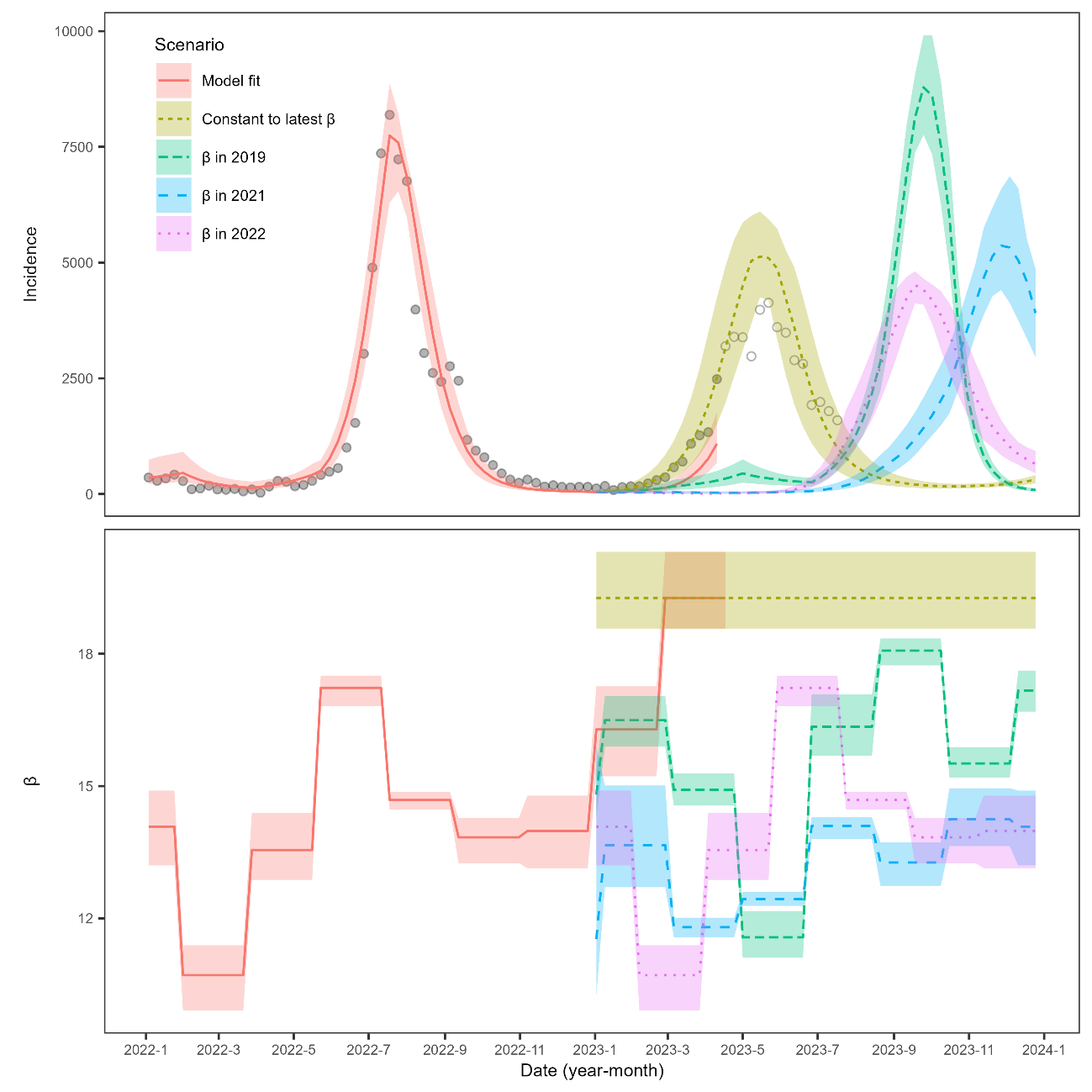


**Figure S3 RSV infection incidence and transmission rate,** $\boldsymbol{\beta}_{\boldsymbol{t}}$ **estimated using the time-series susceptible-infected-recovered (TSIR) model in 2022 and 2023 in Osaka, Japan.** The incidence panel is the replication from Figure 1 in the main text. The $\beta$ panel shows the estimated $\beta_{t}$ from the model fit (red) and the scenario used for corresponding forecasting in 2023: constant to the latest value estimated (constant to latest $\beta_{t}$, gold), (b) the same level of $\beta_{t}$ in 2019 ($\beta_{t}$ in 2019, green), (c) $\beta_{t}$ in 2021 (blue), and (d) $\beta_{t}$ in 2022 (pink). The lines and shades represent the median and 95% confidence interval estimations. The filled dots are the numbers of incidences used for the model fit, and the open dots are those not used. They are calculated as the number of reported cases, $C_{t}$ over the reporting rate estimated, $\delta_{t}$ ($C_{t}/\delta_{t}$).


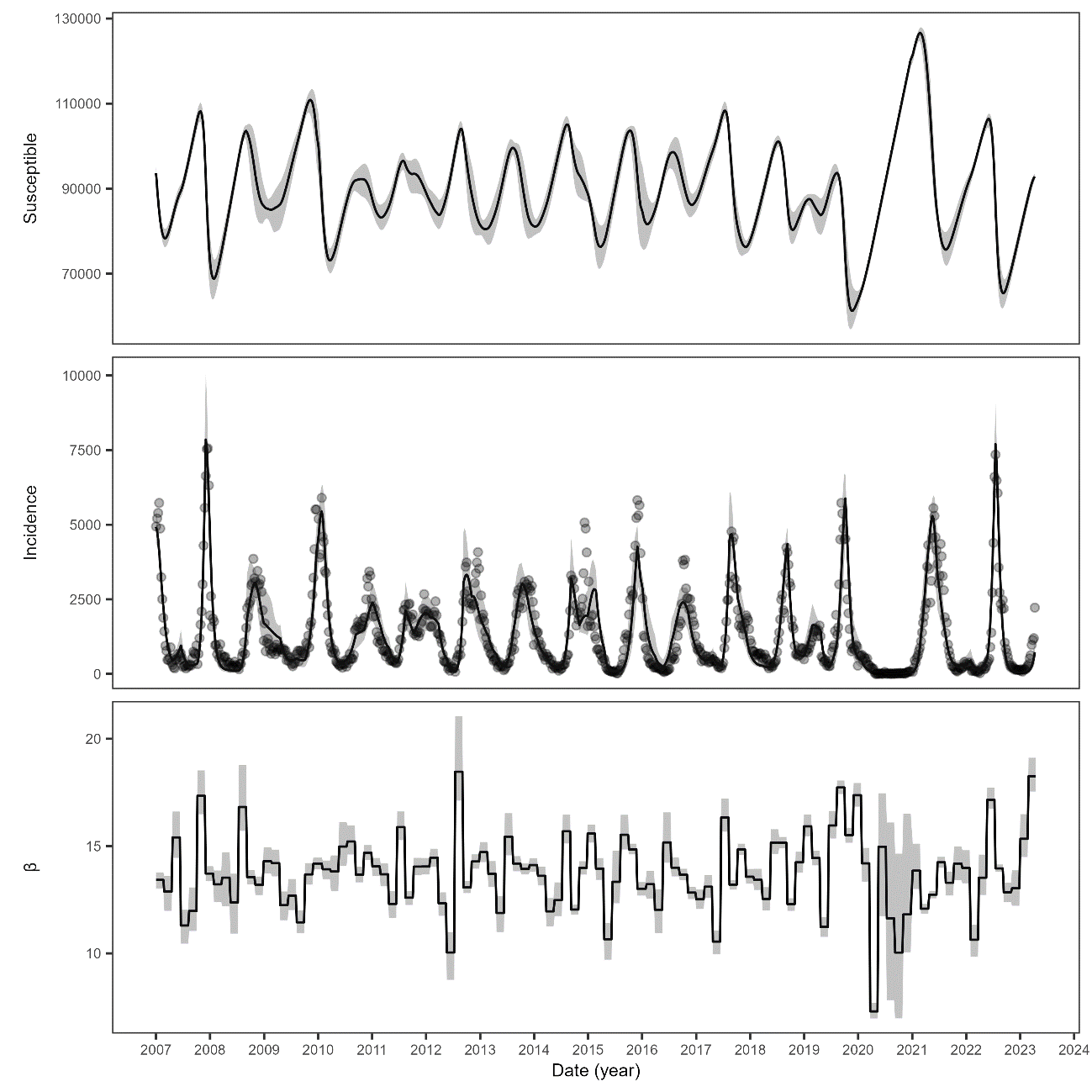


**Figure S4 Sensitivity analysis results: reconstructed number of susceptible individuals and incidences (**$\boldsymbol{S}_{\boldsymbol{t}}$ **and** $\boldsymbol{I}_{\boldsymbol{t}}$**) and estimated transmission rate,** $\boldsymbol{\beta}_{\boldsymbol{t}}$ **using the time-series susceptible-infected-recovered (TSIR) model from 2007 to week 15 in 2023 in Osaka, Japan, with the estimated reporting rate using the smoothing spline method with 8 degrees of freedom.** The lines and shades represent the median and 95% confidence interval estimations. The filled dots are the numbers of incidences used for the model fit. They are calculated as the number of reported cases, $C_{t}$ over the reporting rate estimated, $\delta_{t}$ ($C_{t}/\delta_{t}$).


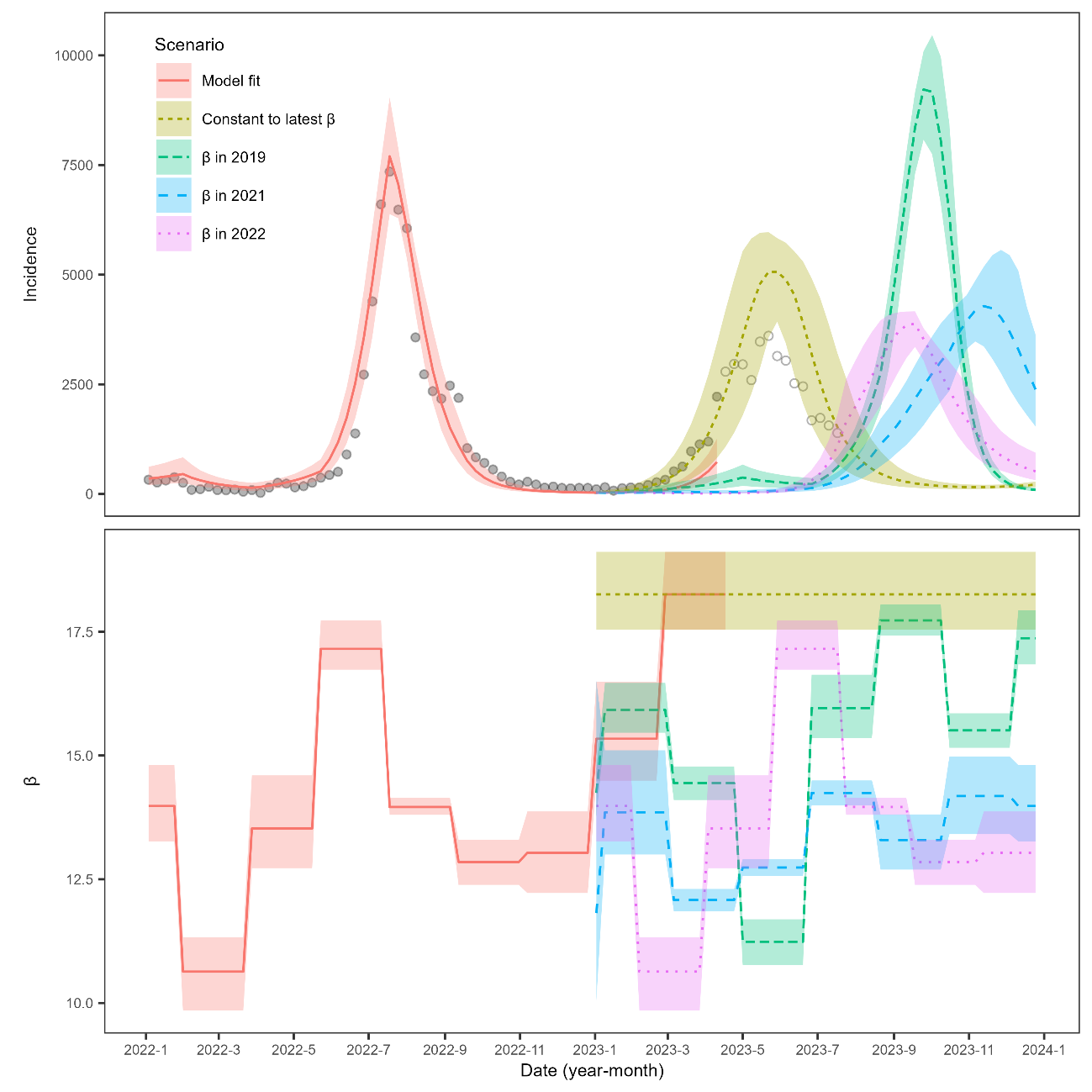


**Figure S5 Sensitivity analysis result: The RSV infection incidence and transmission rate,** $\boldsymbol{\beta}_{\boldsymbol{t}}$ **estimated using the time-series susceptible-infected-recovered (TSIR) model in 2022 and 2023 in Osaka, Japan, with the estimated reporting rate using the smoothing spline method with 8 degrees of freedom.** The $\beta$ panel shows the estimated $\beta_{t}$ from model fit (red) and the scenario used for corresponding forecasting in 2023: constant to the latest value estimated (constant to latest $\beta_{t}$, gold), (b) the same level of $\beta_{t}$ in 2019 ($\beta_{t}$ in 2019, green), (c) $\beta_{t}$ in 2021 (blue), and (d) $\beta_{t}$ in 2022 (pink). The lines and shades represent the median and 95% confidence interval estimations. The filled dots are the numbers of incidences used for the model fit, and the open dots are those not used. They are calculated as the number of reported cases, $C_{t}$ over the reporting rate estimated, $\delta_{t}$ ($C_{t}/\delta_{t}$).


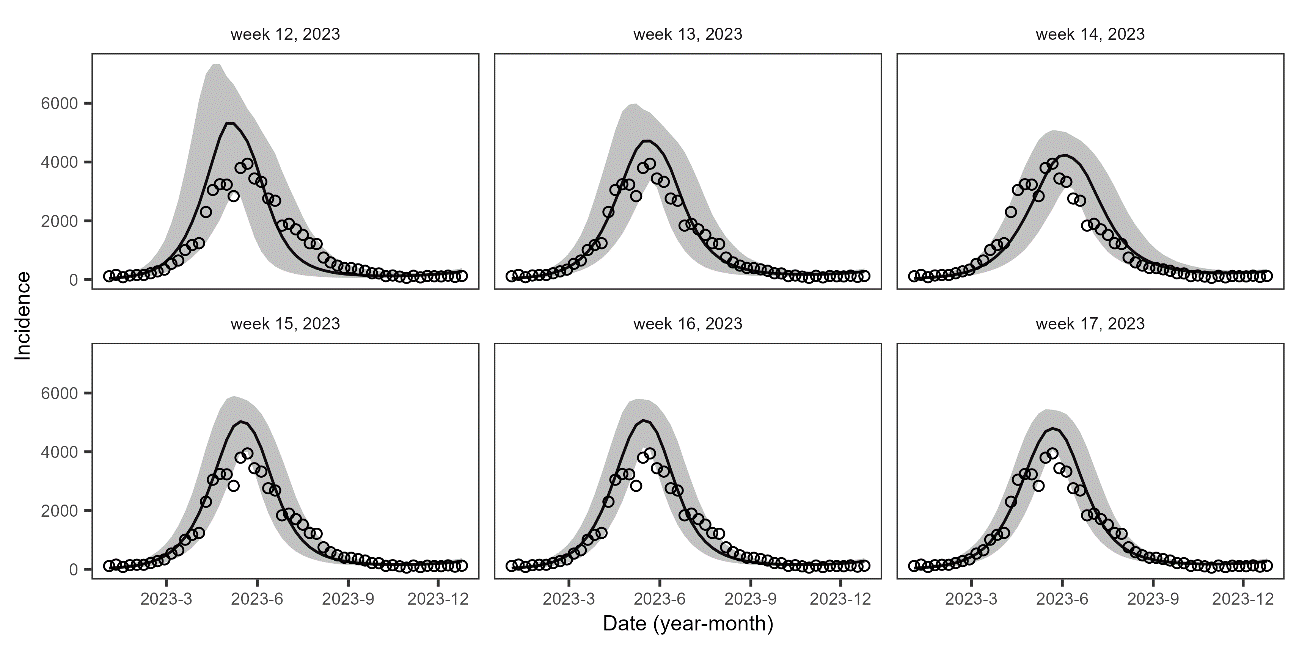


**Figure S6 Forecasting results of RSV infection incidence depending on the cutoff date for observed data period used, assuming the transmission rate in 2023 is constant to the latest estimated β (constant to latest β scenario).** The weeks above each panel indicate the data cutoff time used for β estimation. The vast majority of the observed data was included within the confidence interval (the gray shaded area) in each panel.

References

1. Bjørnstad ON, Finkenstädt BF, Grenfell BT. Dynamics of measles epidemics: Estimating scaling of transmission rates using a Time series SIR model. Ecol Monogr 2002;72:169-184.

2. Finkenstädt BF, Grenfell BT. Time series modelling of infectious diseases: a dynamical systems approach. J R Stat Soc Ser C 2000;49:187-205.

3. Grenfell BT, Bjørnstad ON, Finkenstädt BF. Dynamics of measles epidemics: Scaling noise, determinism, and predictability with the TSIR model. Ecol Monogr 2002;72:185-202.

4. Glass K, Xia Y, Grenfell BT. Interpreting time-series analyses for continuous-time biological models - Measles as a case study. J Theor Biol 2003;223:19-25.

5. Finkenstädt BF, Bjørnstad ON, Grenfell BT. A stochastic model for extinction and recurrence of epidemics: estimation and inference for measles outbreaks. Biostatistics 2002;3:493-510.

6. Chowell G. Fitting dynamic models to epidemic outbreaks with quanti fi ed uncertainty : A primer for parameter uncertainty , identi fi ability , and forecasts. Infect Dis Model 2017;2:379-398.

7. Miyama T, Kakimoto K, Iritani N, et al. Exploring the threshold for the start of respiratory syncytial virus infection epidemic season using sentinel surveillance data in Japan. Front Public Heal 2023;11: 1062726.
